# Factors predicting admission of psychiatric emergency contacts after presenting to the emergency department: results of a regression analysis

**DOI:** 10.1186/s12991-022-00421-2

**Published:** 2022-11-09

**Authors:** Heribert Kirchner, Martin Schaefer, Heiko Ullrich, Nik Hulsmans, Georg Juckel, Patrick Brzoska, Frank-Gerald Bernhard Pajonk

**Affiliations:** 1 Faculty of Health, School of Medicine, University of Witten/Herdecke, Witten, Germany; 2 Department of Psychiatry, Psychotherapy, Psychosomatics and Addiction Medicine, Evang. Kliniken Essen-Mitte, Essen, Germany; 3 Department of Psychiatry and Psychotherapy, Charité–Universitätsmedizin Berlin, Campus Charité Mitte, Berlin, Germany; 4 Department of Psychiatry, Psychotherapy and Psychosomatics, Kreisklinikum Siegen, Hospital, Siegen, Germany; 5 Department of Psychology, University of Siegen, Siegen, Germany; 6 Department of Psychiatry, Psychotherapy and Preventive Medicine, LWL University Hospital, Ruhr University Bochum, Bochum, Germany; 7 Zentrum Isartal Am Kloster Schäftlarn, Schäftlarn, Germany; 8 Department. of Psychiatry and Psychotherapy, Technical University Munich, Munich, Germany

**Keywords:** Psychiatric emergency, Regression analysis, Emergency department, Predictor, Admission

## Abstract

**Background:**

Psychiatric emergency patients have great relevance in the interdisciplinary emergency department. Emergency physicians in this setting often have to make decisions under time pressure based on incomplete information regarding the patient’s further treatment. The aim of this study was to identify possible predictors associated with an increased likelihood of inpatient psychiatric admission.

**Methods:**

A retrospective cross-sectional study of all psychiatric emergency contacts in an interdisciplinary emergency department (ED) of a general hospital in a large German city was conducted for 2015. A binary regression analysis was performed to identify possible predictors.

**Results:**

In 2015, a total of 21421 patient contacts were reported in the emergency department, of which 1733 were psychiatric emergencies. Psychiatric emergency was the fourth most common cause presenting to the ED. The most common diagnosis given was mental and behavioral disorders due to the use of psychotropic substances (F1). Factors associated with an increased probability of inpatient psychiatric admission were previously known patients, patients under a legal care order (guardianship), and previous outpatient medical contact. No association for gender or age was found. Data demonstrated a negative relationship between a neurotic, stress-related and somatoform disorder diagnosis and admission.

**Conclusions:**

The present study shows some significant characteristics associated with an increased likelihood of emergency admission. Independent of the health care system, the predictors found seem to be relevant with regard to the probability of admission, when compared internationally. To improve the treatment of patients in emergency units, these factors should be taken into account.

## Background

The relevance of interdisciplinary emergency departments (ED) as care units has increased significantly during the last two decades. In 2019, approximately 21 million patients were treated in German emergency departments, with an estimated annual growth rate of approximately 4–5%, of which approximately 5–10% were psychiatric emergencies [1–4]. Psychiatric emergency patients become increasingly important in this setting [5]. In the Anglo-American region, a continuous increase in psychiatric cases in EDs has been described for the last two decades [6], and an increase of approximately 16% between 2000 and 2010 was also found in Germany [1].

Patients arrive at the ED through emergency medical services, ambulance services, the police, or individuals approach the ED themselves with or without referral [1, 2, 7, 8]. There are typical emergency indications (see Table 1) named in the German guideline “Emergency Psychiatry” [9], that may indicate an increased likelihood of inpatient admission. The absolute and relative psychiatric emergencies listed in Table 1 differ with regard to their need for immediate action. The “absolute psychiatric emergency” usually requires immediate diagnostic and/or treatment, which is usually not the case for relative psychiatric emergencies. According to the German guideline for emergency psychiatry, psychiatric emergencies are defined as “a medical situation in which the acute onset or exacerbation of an existing psychiatric disorder leads to an immediate threat to the life and health of the affected person and/or his or her environment and requires immediate diagnosis and/or therapy [9].”

**Table 1:** Psychiatric emergency criteria (German Guideline—DGGPN 2019)

Absolute psychiatric emergency	Relative psychiatric emergency
-Suicide attempt -Concrete suicide ideas/plans -Severe intoxication -Severe state of arousal -Aggressiveness/violence caused by mental disorder -Delirium	-Confusion -Withdrawal without delirium -Suicidality without intention -Anxiety and panic disorder -Acute adjustment reaction and psychosocial dysfunction

Table 1 lists all important absolute and relative psychiatric emergencies according to the German guideline for emergency psychiatry (2019)

In the ED, in addition to the initial diagnosis, the decision about the patient's need for further treatment must be made quickly by means of an assessment, even if little information is available [6, 10].

To date, there are only unspecific criteria under which conditions emergency psychiatric patients should be admitted as inpatients, in part because the aims and methods of individual studies have been inconsistent [7, 11–16]. However, despite differences in study designs and country-specific differences in health care systems, some predictors are described repeatedly. These include the presence of suicidality and a diagnosis of schizophrenia [11, 12, 17–20], as well as the presence of aggressiveness, impulsivity, and danger to others [14, 16, 20, 21]. In contrast, diagnoses such as anxiety panic disorder are more likely not to result in inpatient admission [7]. The particular mode of transport of the patient to the ED (via ambulance and/or police) also appears to have an impact on the likelihood of admission [7, 11, 13, 22]. In addition to these factors, age and referral by a general practitioner are also reported as additional predictors [11, 14, 15, 19, 23, 24]. Considering that previous studies on the factors associated with the admittance of emergency psychiatric patients to hospitals have been inconsistent, the purpose of the present secondary data analysis was to add to the existing body of research by examining how recent data from a large German hospital compares to previous investigations. By doing so, we draw conclusions for the further development of algorithms in psychiatric emergency care, both nationally and internationally.

## Methods

A retrospective evaluation of all psychiatric emergency contacts in the ED of the Kreisklinikum Siegen, a hospital in a large German city, was conducted. In the study period (01.01.2015-31.12.2015), Kreisklinikum Siegen had 556 beds and 11 specialized departments, including a psychiatric department with 140 beds, and was responsible for the entire district of 280,000 inhabitants. The ED is part of the emergency medical care of the city of Siegen. Almost all psychiatric emergencies are presented by emergency physicians, rescue service and police. In addition, it is possible for patients to present themselves on their own initiative, with or without a referral from a physician, at any time of the day on all days of the week.

The ED of the examined hospital is the only one in the city with a psychiatric department at a general hospital, covering all potential psychiatric emergency patients in the entire urban region. All patients with a primary psychiatric diagnosis and a minimum age of 18 years who presented at the ED were included in the study. These patients were examined by a consultant in psychiatry and psychotherapy, who also made the diagnosis (according to ICD-10) and decided on further treatment and, if necessary, inpatient admission. Data collection was performed by a consultant psychiatrist and psychotherapist working in the psychiatric hospital. Age, sex, diagnosis, and, if present, an emergency psychiatric syndrome according to the German S2k guideline “Emergency Psychiatry” were documented [9]. The following additional data were recorded: date and time of presentation, legally initiated deprivation of liberty measures due to a mental disorder, outpatient or inpatient psychiatric pretreatment in the hospital, performance of medical clearance (basic examination as laboratory tests and ECG), referral by a general practitioner, and route of access to the ED.

Statistical analysis was conducted using SPSS 25. Multivariable regression analysis was performed to determine predictors of a patient's hospitalization. The following factors were included in the analysis: age, sex, day of the week, time of day, month, previous outpatient or inpatient psychiatric treatment at the investigated hospital, admission to the hospital due to legal measures, referral by a physician, route of access (emergency medical and ambulance service, police, self-initiated), emergency psychiatric syndrome, existence of suicidality, performance of ECG, laboratory tests and imaging (CT or MRI), main diagnosis according to International Classification of Diagnosis (ICD-10; F0–F7), and use of drugs. Since the dependent variable was a binary (categorical) variable (stay: yes/no), binary logistic regression was chosen to analyze the data. According to the omnibus test, the regression model had significant explanatory power with a *p* value < 0.001. Nagelkerke R-squared of 0.747 indicated a high explanatory power of the model. Because the regression analysis performed on all parameters collected was a very large model with many non-significant predictors, the analysis comprised two steps. In the first step of the regression analysis, we examined all recorded parameters of the study shown in Table 2. Subsequently, in the second step, we only included predictors significant at *p* < 0.1 in the first step that were predictive of an increased probability of admission.

**Table 2:** First regression analysis of all variables

Variable	Category	Regressions coefficient B	Odds ratio	*p*-value	CI (95%)
Age range (Reference: < 25 years)	25–60	.051	1.052	.834	0.65–1.69
	> 60	.356	1.427	.309	0.72–2.83
Patient known (pretreated at the hospital) (Reference: no)	Missing	− 17.609	.000	1	1.75–3.76
	Yes	.942	2.566	.000	
Sex (Reference: female)	Male	.181	1.198	.322	0.84–1.71
Weekday (Reference: Sunday)	Monday	.105	1.111	.744	0.59–2.09
	Tuesday	.230	1.258	.471	0.67–2.35
	Wednesday	.456	1.578	.164	0.83–3.00
	Thursday	− .107	.899	.751	0.47–1.73
	Friday	− .304	.738	.362	0.38–1.42
	Saturday	− .169	.844	.625	0.43–1.67
Time of arrival (Reference: 0:00–3:49)	04:00–7:59	− .499	.607	.300	0.24–1.56
	08:00–11:59	− .408	.665	.258	0.33–1.35
	12:00–15:59	− .147	.863	.675	0.43–1.72
	16:00–19:59	− .167	.846	.643	0.42–1.71
	20:00–23:59	.157	1.170	.883	0.58–2.38
Legal status (Reference: voluntary)	Involuntary by psychkg	3.433	30.962	.000	7.96–120.37
	Involuntary by BtG	3.417	30.469	.000	4.49–206.78
	Missing	3.659	38.839	.091	0.56–2712.94
Referral to hospital (Reference: no)	Yes by general physician	1.452	4.271	.001	1.87–9.74
	Yes by emergency physician	− 2.110	.121	.000	0.05–0.31
	Missing	− 1.733	.177	.105	0.02–1.44
Mode of transportation (Reference: by foot)	Ambulance	− .418	.658	.065	0.42–1.03
	Police	.670	1.955	.215	0.68–5.64
	Others	− .760	.468	.624	0.02–9.74
	Missing	− 2.158	.116	.029	0.02–0.80
Psychiatric emergency according to the German guideline. (Reference: no)	Yes	1.048	2.851	.000	1.94–4.20
	Missing	− 1.291	.275	.517	0.01–13.58
Diagnosis according to ICD-10 F0 (Reference: no)	Yes	− .782	.458	.377	0.08–2.59
	Missing	− 22.851	.000	.998	0
Diagnosis according to ICD-10 F1 (Reference: no)	Yes	− 2.252	.105	.001	0.03–0.39
	Missing				
Diagnosis according to ICD-10 F2 (Reference: no)	Yes	− 1.785	.168	.044	0.04–0.64
	Missing				
Diagnosis according to ICD-10 F3 (Reference: no)	Yes	− 2.381	.092	.000	0.02–0.35
	Missing				
Diagnosis according to ICD-10 F4 (Reference: no)	Yes	− 3.817	.022	.000	0.01–0.09
	Missing				
Diagnosis according to ICD-10 F5 (Reference: no)	Yes	− 4.128	.016	.009	0.00–0.36
	Missing				
Diagnosis according to ICD-10 F6 (Reference: no)	Yes	− 1.320	.267	.066	0.07–1.09
	Missing				
Diagnosis according to ICD-10 F7 (Reference: no)	Yes	− 1.710	.181	.219	0.01–2.76
	Missing				
Suicide attempt X84 (Reference: no)	Yes	21.075	> 1 Mrd	.998	0
	Missing	31.730	> 1 Mrd	.999	0
Intoxication according to ICD-10 T36–T50 (Reference: no)	Yes	1.634	5.125	.170	0.50–52.89
	Missing	− 34.218	.000	.999	0
Internal additional diagnosis (Reference: no)	Yes	− 2.484	.083	.000	0.04–0.20
	Missing	− 11.734	.000	.999	0
Neurological additional diagnosis (Reference: no)	Yes	− .182	.834	.757	0.26–2.64
	Missing				
Surgical additional diagnosis (Reference: no)	Yes	− .047	.954	.925	0.36–2.56
	Missing				
Psychopharmaca administered at ER (Reference: no)	Yes	.376	1.457	.784	0.09–21.60
	Missing	15.371	> 4 Mio	.999	0
Benzodiazepine administered at ER (Reference: no)	Yes	− .628	.534	.558	0.07–4.36
	Missing				
High potency antipsychotics administered at ER (Reference: no)	Yes	18.374	> 9 Mrd	.999	0
	Missing				
Low potency. antipsychotica administered at ER (Reference: no)	Yes	− .989	.372	.460	0.03–5.14
	Missing				
Antidepressiva administered at ER (Reference: no)	Yes	− 49.524	.000	.999	0
	Missing				
Internal medications administered at ER (Reference: no)	Yes	− 1.354	.258	.189	0.03–1.95
	Missing				
Other medications administered at ER (Reference: no)	Yes	1.345	3.840	.283	0.33–44.83
	Missing				
ECG at ER (Reference: no)	Yes	1.382	3.982	.000	2.47–6.41
	Missing				
Laboratory at ER (Reference: no)	Yes	2.789	16.262	.000	9.93–26.64
	Missing				
Radiological examination at ER (Reference: no)	Yes	− .191	.826	.664	0.35–1.96
	Missing				

Table 2 includes all variables examined in the study related to emergency psychiatric patients presented in the ER during 2015

## Results

**Fig. 1:**
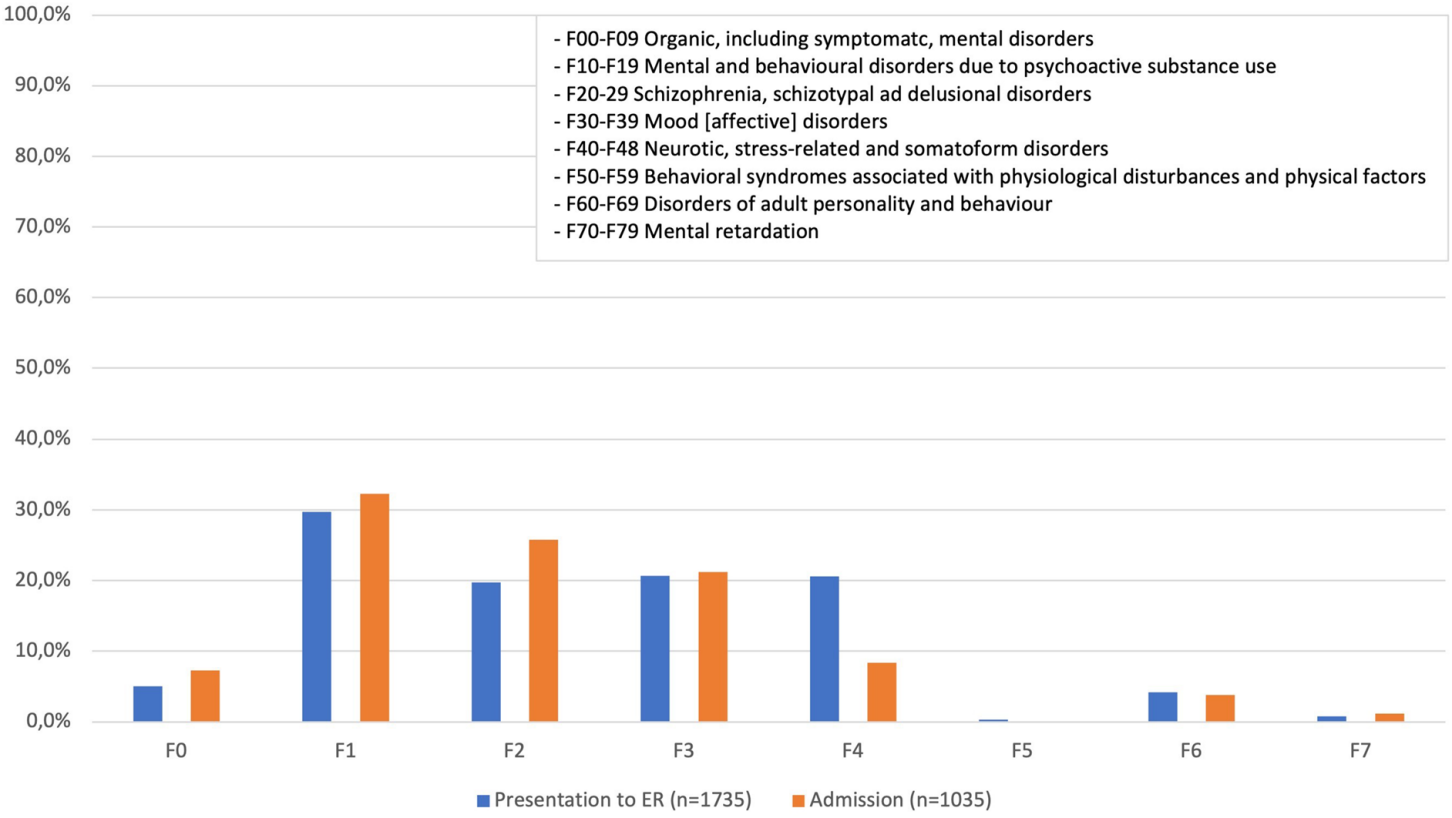
Shows the share of each diagnosis in the total number of patients presenting to the ER (blue columns). Furthermore, the red columns show the percentage of inpatient admissions corresponding to diagnoses F0–F7

In 2015, there were a total of 21421 patient contacts in the emergency department, of which 1733 (8.1%) were psychiatric emergencies. Psychiatric emergency was the fourth most common cause presenting to the ED after internal medicine, surgery, and neurology emergencies. The mean age of psychiatric patients was 42.7 years (SD = 17.1), and the proportion of male patients was 51.8% (*n* = 898). After presentation to the ED, almost 60% of emergency psychiatric patients were admitted as inpatients. The most common diagnosis given was mental and behavioral disorders due to the use of psychotropic substances (F1). Diagnoses from the categories schizophrenia, schizotypal and delusional disorders (F2), mood [affective] disorders (F3), and neurotic, stress-related and somatoform disorders (F4) did not differ much in frequency. Patients with a mental and behavioral disorder caused by psychotropic substances were also the most frequently admitted (see Fig. 1).

Before conducting the regression analysis some of the outpatients and inpatients had to be excluded. The initial dataset comprises *N* = 1898 cases. Of these, a total of *n* = 163 cases was excluded due to missing values. In the regression analysis, *n* = 2 cases were further excluded due to a Cook Distance significantly  > 1. The final sample size thus comprises *N* = 1733.

The results of the final regression analysis to identify possible predictors associated with an increased likelihood of admission are shown in Table 3. Not all of the 32 parameters initially included in the model remained in the final regression model. Among others, the involuntary presentation of patients was strongly associated with admission (OR = 25.9; 95%-CI 8.9–75.7), performed laboratory (OR 15.0, 95%-CI 9.4–24.1) and ECG examinations (OR 3.6, 95% CI 2.3–5.8), and a referral by a physician (OR = 5.0, 95%-CI 2.3–10.7) were also associated with an increased probability of admission. If the criteria for a psychiatric emergency according to the German guideline are met, the probability of being admitted as an inpatient also increases (OR 2.8, 95%-CI 1.9–4.1). Patients who had already been psychiatrically treated in the hospital earlier were also more likely to be admitted (OR 2.5, 95%-CI 1.7–3.7).

**Table 3:** Final regression analysis of admission predictors at the ER

Variable	Regression coefficient B	Odds ratio	*p*-value	CI (95%)
Admission against patients’ will (yes or no)	3.255	25.932	.000	8.864–75.865
Laboratory at ER (yes or no)	2.731	15.080	.000	9.401–24.192
ECG at ER (yes or no)	1.303	3.681	.000	2.322–5.836
Referral to hospital by physician (yes or no)	1.615	5.028	.000	2.346–10.780
Psychiatric emergency according to the German guideline (yes or no)	1.056	2.875	.000	1.994–4.145
Patient known (pretreated at the hospital yes or no)	0.921	2.511	.000	1.775–3.756
Referral by emergency physician (yes or no)	− 1.749	.174	.000	.078–.390
Mode of transportation (ambulance/EP) (yes or no)	− .523	.539	.012	.394–.893
Mental and behavioral disorders due to psychoactive substance use (ICD-10: F1) (yes or no)	− 1.641	.194	.000	.086–.435
Schizophrenia, schizotypal and delusional disorders (ICD-10: F2) (yes or no)	− 1.200	.301	.006	.129–.705
Mood [affective] disorders (ICD-10: F3) (yes or no)	− 1.764	.171	.000	.075–.392
Neurotic, stress-related and somatoform disorders (ICD-10: F4) (yes or no)	− 3.174	.042	.000	.018–.096
Behavioral syndromes associated with physiological disturbances and physical factors (ICD-10: F5) (yes or no)	− 3.179	.042	.028	.002–.712

Table 3 lists all relevant predictors in terms of OR and significance

There was no increased probability of admission for any principal diagnosis. Age and gender were also not associated with an increased probability of admission. However, a significantly decreased probability of admission was found for neurotic, stress-related and somatoform disorders.

## Discussion

The aim of this study was to identify predictors for an increased probability of admission of inpatient psychiatric emergency patients after presentation to an ED. To date, there has been no further development of clear SOPs although there have been few international studies on this issue since the 1980s, two of which were from Germany.

One reason may be the lack of data and inconsistent results of previous studies because of the lack of development and implementation of SOPs for psychiatric emergency patients.

The patients we studied largely matched those of other studies [1, 2, 8, 12, 15, 16, 19, 25–27] in terms of age, diagnostic spectrum, and admission rate.

Consistencies in predictors of inpatient psychiatric admission include prior psychiatric treatment [7, 11, 28], presence of legally initiated admissions against the patient's will [7, 21], and physician referral [7, 12, 21, 22]. The psychiatric emergency syndromes from the German guideline Emergency Psychiatry [9] are also predictive, supporting the validity of the syndromes.

In contrast to other studies, none of the ICD-10 diagnoses were associated with an increased probability of admission. Some of these studies found a diagnosis of schizophrenia, schizotypal and delusional disorders (F2), or mood [affective] disorders (F3) as predictors [11–13, 16]. Although neurotic, stress-related, and somatoform disorders (F4) account for a significant proportion of psychiatric patients in the ED, they appear to be associated with a decreased likelihood of inpatient admission. This has also been found by other studies [7, 12, 28]. In our study, suicidality was not a predictor of inpatient admission, although this has been found in most other studies. This may be due to the comparatively low documented frequency of suicide attempts or existing acute suicidality (3%) among patients in our study. The reason for this is probably the incomplete documentation of suicidality in the emergency department and, because of the study design with anonymization, the impossibility of combining different patient records from the emergency physician service, emergency department, and psychiatric hospital. Differences between our study and most other studies were also found with regard to the factors age and gender. In other studies, older age was a predictor [11, 14, 15, 19, 22]. Regarding gender, the results are inconsistent. In an Italian study, a higher probability was found for men [12], while in an American study, the probability was higher for women [19]. Factors that have been shown to be predictive in other studies, such as the presence of aggressiveness, apathy, psychotic perception, thought disorder [7, 11, 16, 28], or homelessness [19], were not examined.

A limitation of our study was the retrospective design. Therefore, maybe in some cases it was a challenge to assess the severity of clinical syndromes, which may have led to a lack of clarity in the distinction between the presence or non-presence of a psychiatric emergency. Due to anonymization of data collection, it was not possible to merge different data sources from prehospital emergency medicine, EDs, and psychiatric hospitals. Because of that, prehospital treatment in another clinic could not be excluded with certainty. Consequently, in some cases possible conclusions about patients with a first psychiatric illness could have been over- or underestimated. The results are data from a single ED and are therefore not representative of Germany.

## Conclusions

The findings of our study with respect to factors associated with the admittance of psychiatric emergency patients to hospitals are similar to results from other national and international investigations, despite differing health care systems.

Based on our and previous investigations, the following determinants of admittance can be identified: inpatient psychiatric admissions are primarily patients with prior outpatient or inpatient psychiatric treatment, with a physician referral, and with treatment on a legal basis against the patient’s will.

Independent of the health care system, the predictors found seem to have a clear significance with regard to the probability of admission, even when compared internationally. For this reason, it would be desirable for subsequent studies to take a more differentiated look at these predictors in order to develop SOPs for more efficient patient care.

## Data Availability

The datasets generated during and/or analysed during the current study are available from the corresponding author on reasonable request.
